# Supplementary material for: Clinical Features and Outcomes of Primary Cutaneous Peripheral T-Cell Lymphoma, Not Otherwise Specified, Treated with CHOP-Based Regimens
Source: Cancers (Basel). 2025 May 15;17(10):1673. doi: 10.3390/cancers17101673 (PMC12109765; doi:10.3390/cancers17101673)
Supplement: Supplementary file 1 [file cancers-17-01673-s001.zip › cancers-3603795-supplementary.pdf]

Supplementary Table S1. Summary of clinicopathological characteristics for patients with primary cutaneous peripheral T-cell lymphoma, not otherwise specified

Characteristics	Primary cutaneous PTCL-NOS (N = 15)
Age	
≤60	10 (66.67%)
>60	5 (33.33%)
Sex	
Female	11 (73.33%)
Male	4 (26.67%)
Stage	
T1	5 (33.33%)
T2	4 (26.67%)
T3	6 (40.00%)
B_symptoms	
No	13 (86.67%)
Yes	2 (13.33%)
B2_microglobulin	
Normal	11 (73.33%)
Elevated	4 (26.67%)
Serum LDH	
Normal	11 (73.33%)
Elevated	4 (26.67%)
Ki67	
<80%	9 (60.00%)
≥80%	6 (40.00%)
CD20	
Negative	10 (66.67%)
Positive	5 (33.33%)
CD30	
Negative	9 (60.00%)
Positive	6 (40.00%)
PD_1	
Negative	12 (80.00%)
Positive	3 (20.00%)
Immunophenotype	
CD4+/CD8-	8 (53.33%)
CD4-/CD8+	1 (6.67%)
CD4-/CD8-	2 (13.33%)
CD4+/CD8+	4 (26.67%)
First-line therapy	
Chemotherapy	3 (20.00%)
Chemo+ Chidamide	7 (46.67%)
Chemo+ BV	2 (13.33%)
Chemo+ Lipo_MIT	2 (13.33%)
Chemo+ Radiotherapy	3 (20.00%)
ASCT	2 (13.33%)
Chemo+ Surgery	3 (20.00%)
